# Physiological Responses of Cucumber Seedlings to Different Supplemental Light Duration of Red and Blue LED

**DOI:** 10.3389/fpls.2021.709313

**Published:** 2021-07-12

**Authors:** Shuya Wang, Hua Fang, Jianming Xie, Yue Wu, Zhongqi Tang, Zeci Liu, Jian Lv, Jihua Yu

**Affiliations:** ^1^College of Horticulture, Gansu Agricultural University, Lanzhou, China; ^2^Gansu Provincial Key Laboratory of Arid Land Crop Science, Gansu Agricultural University, Lanzhou, China

**Keywords:** LED, supplemental light duration, cucumber, growth, photosystem, photoreceptor

## Abstract

Normal development of plants is inhibited by inadequate light in winter in greenhouses in Northwest China. Growth lamps, using light-emitting diodes (LEDs) with red blue light (7R2B), were used to supplement daylight for 1, 2, and 3 h. Seedling growth, photosynthesis, and photosynthetic product; the Calvin cycle key and sugar metabolism-related enzymes and their encoding genes; and the light signal sensing regulation of key gene expression were studied in greenhouse cucumbers under three treatments to determine the best supplemental light durations to enhance cucumber cultivation in greenhouses in winter. Treatment with LED red and blue light for 3 h significantly promoted the growth and development of cucumbers, root growth, and dry matter accumulation. It improved the photosynthetic rate, photosynthetic pigment content, and light energy utilization efficiency in cucumbers. Supplementation with red and blue LED light for 3 h upregulated the expression levels of key genes encoding the Calvin cycle and enzymes related to sugar metabolism in cucumber leaves, which promoted the synthesis and accumulation of photosynthates. The expression levels of phytochrome B, cryptochrome 1, and hypocotyl 5 in the cucumber leaves were also significantly upregulated after 3 h of light supplementation. Combined LED red and blue light for 3 h should be used to supplement natural light to enhance the cucumber cultivation in greenhouses in winter.

## Introduction

Light is a critical factor influencing the various stages of plant growth and development. It is also the basis of the energy metabolism of plants. The ability of plants to absorb and assimilate CO_2_ decreases when there is a lack of light during the plant growth (Fankhauser and Chory, 1997; Deng and Quail, 1999). This results in a decrease in photosynthetic products and eventually leads to a reduced crop growth. In Northwest China, the sunshine hours in winter are shortened and accompanied by continuous snow. Lighting time was reduced to approximately 7 h in the greenhouse, and the average period of light intensity per day greater than 30,000 lx was only 4.5 h (Qi et al., 2016). In addition, factors such as materials covering the greenhouse, aging of the glass, and dust covering lead to a poor light environment in the greenhouse. This results in plant spindling, serious flower and fruit dropping, slow fruit development, and frequent occurrence of pests and diseases (Dorais et al., 1996; Massa et al., 2008; Cockshull et al., 2015). Artificial supplementation of light is an effective way to alleviate insufficient light when cultivating vegetables in winter in Northwest China. Improving light conditions promotes the plant growth and development, enhances the vegetable quality, and increases the yield (Jie et al., 2003; Dougher and Bugbee, 2004; Qian and Kubota, 2009). Light-emitting diodes (LEDs) provide low heat sources in diversified devices that are highly efficient and advantageous in terms of environmental protection. LED lighting can be adjusted for light quality, light intensity, and photoperiod according to specific needs, and most plants can complete their entire growth and development under the LED lights. This makes LEDs the ideal source for greenhouse lighting.

The absorption spectra of photosynthetic pigments in plant leaves are mainly red and blue-violet light. Red light is required for photosynthesis. Blue light is important for physiological processes such as chloroplast development, chlorophyll synthesis, and stomatal opening (Senger and Briggs, 1981; Sæbø et al., 1995). Red and blue light can induce the highest rates of photosynthesis, and the combination of red and blue light provides the highest photon efficiency (Nelson et al., 2014). Many studies have reported red and blue LED lights as the regulators of plant growth and development (Liu et al., 2018; Izzo et al., 2020; Zhang J. et al., 2020). Our previous study found that supplementing red and blue LED light (7R2B) at night in winter can significantly improve the growth, fruit quality, and yield of tomatoes and zucchini, thereby increasing income for farmers (Wang et al., 2018, 2020). Previous research showed that adding red light to a greenhouse environment increases the leaf area and the distribution of dry matter in the leaves of grapes (Kong et al., 2006). Blue light, which is provided during LED supplementation, increases the contents of plant chlorophyll a/b, affects the photomorphogenesis of plants, and promotes the opening of plant stomata (Baroli et al., 2008). Another study showed that the supplementation with an appropriate ratio of blue and red light in LED phototherapy can increase the dry weight, number of leaves, and leaf area of tomatoes. It also promotes the growth of tomatoes by improving the efficiency of photosynthetic light utilization and regulating the root vitality under natural sunlight conditions in the greenhouse (Gomez and Mitchell, 2015; Xu et al., 2016; Paponov et al., 2020). In addition, supplementation with LED phototherapy using an appropriate ratio of blue and red light may promote the growth and nutrient absorption of lettuce, and makes some ornamental plants more compact, with increased stem diameters and chlorophyll content (Randall and Lopez, 2014; Pinho et al., 2016; Yan et al., 2020). Red and blue LED light can significantly increase the chlorophyll content, the net photosynthetic rate, and the stomatal conductance of the leaf in cucumbers and lettuce and improve their photosynthetic capacity (Hogewoning et al., 2010; Hernández and Kubota, 2016; Wang et al., 2016). Red-blue LED treatment significantly improves the electron transfer rate, the photochemical efficiency coefficient (ΦPS II), the photochemical quenching (QP), and the efficiency of capturing excitation energy (Fv′/Fm′) in the open Photosystem II (PS II) reaction center (Liu, 2012), whereas single light quality red and blue LEDs inhibits the PS II efficiency (Wang et al., 2009).

Plant light morphology is mediated mainly by the unique photoreceptor phytochromes (PHYA, PHYB) and cryptochromes (CRY1, CRY2, and CRY3) (Hogewoning et al., 2010). Phytochromes are sensitive to red light, whereas cryptochromes are sensitive to blue light (Kadir et al., 2017). Thus, phytochromes and cryptochromes work together by interacting in both a light-dependent and a light-interdependent manner (Guo et al., 1998; Mas et al., 2000). HYPOCOTYL5 (HY5) is a downstream optical signal regulator in the phytochrome, which plays a positive role in regulating photomorphogenesis (Oyama et al., 1997; Osterlund et al., 2000; Lau and Deng, 2012). A previous study showed that LED phototherapy using a blue and red light combination upregulated the expression level of HY5 in tomatoes and increased the content of lycopene by inducing the expression of PHYA and PHYB (Xie et al., 2019). CRY1 plays a crucial role in Arabidopsis in regulating blue light, which inhibits the hypocotyl elongation (Ahmad and Cashmore, 1993). However, research into the role of PHY, CRY, and HY5 as mediators of the growth and development of cucumbers with red and blue LED lighting is not extensive.

The cucumber (*Cucumis sativus* L.) is one of the main crops cultivated in greenhouses. In this experiment, red and blue LEDs (7R2B) were used as supplementary light sources for plant growth. This study was designed to investigate the effects of different times of supplementary light (1, 2, and 3 h at the end of daylight) on the plant growth, photosynthesis, the Calvin cycle, sugar metabolism, and the influence of light signal sensing regulation of key gene expression in cucumber seedlings in greenhouses. This study also investigated the best times to supplement light for cucumber cultivation in greenhouses in Northwest China. The results provide a solid theoretical foundation for using PHY, CRY, and HY5 in achieving growth and physiological changes under supplemental LED red and blue light in greenhouse cucumber seedlings.

## Materials and Methods

### Plant Material and Growth Conditions

Cucumber (*C. sativus* “Bote 209,” Tianjin Derit Seeds Company Ltd., Tianjin, China) seeds were soaked in 55°C water for 15 min, soaked in clean water for 10 h, and then spread evenly in a petri dish covered with wet filter paper. The petri dish was placed in a constant temperature incubator at 26°C, with a humidity of 75%, for 48 h under dark conditions to promote germination. Seeds showing reliable germination were sown in a 50-hole tray, which contained seedling substrate (Gansu Luneng Agricultural Science and Technology Co., Ltd., Gansu, China). Temperatures were maintained at 26°C during the day and 18°C at night.

### Light Treatments and Experimental Design

**FIGURE 1:**
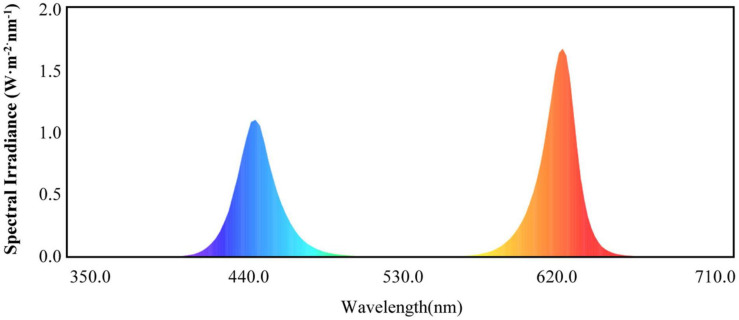
Red and blue (7R2B) LED plant growth light spectrum.

Light-emitting diode lights (HY-115 cm-36 LED plant growth lamps with 3W-RB′ red and blue (7R2B) light, Shenzhen Houyi Energy Saving Technology Company Ltd., Shenzhen, China) had a power rating of 108 W and an illumination intensity of ≤19,130 lx. The LED spectrum is shown in Figure 1. Cucumber seedlings with the same growth levels were selected when the fourth leaf was established. These seedlings were transplanted into pots with cultivation substrate in the greenhouse. Four treatments were used in the study: control, T1, T2, and T3. The control treatment was only natural light; the T1 treatment used LEDs to supplement the light for 1 h when the natural light ended; the T2 treatment used LEDs to supplement the light for 2 h when the natural light ended; and the T3 treatment used LEDs to supplement the light for 3 h when the natural light ended. The treatments were arranged in a completely randomized design with three replicates. Each experimental unit consisted of 30 seedlings, with a total of 90 seedlings per treatment. At 0, 15, and 30 days after treatment, 10 plants were randomly collected from each treatment for the growth data analysis. After 30 days of treatment, at the end of each supplemental light treatment in the evening, 30 plants from each treatment were randomly sampled for physiological and genetic data analyses.

### Determination of Plant Growth

Plant height was defined as the height from the base of the cucumber stem to the growing point. Stem diameter was measured at 1 cm above the basal part of the stem using Vernier calipers. All true leaves with a longitudinal diameter greater than 5 cm were counted. The third leaf from the top of the cucumber was used for leaf measurements. The length (L—the distance from petiole base to tip) and width (W—the maximum width of pulse vertical) of the leaf were measured using a ruler. For the measurement and calculation of leaf area, Xue et al. (2006) were referred. The angle between the petiole and the third leaf from the growth point on the main stem was measured with a protractor. The roots from six plants from each treatment were cleaned and dried, and the aboveground and underground parts were separated (stem and root). The fresh weight was recorded. The samples were dried at 105°C for 30 min and, thereafter, at 80°C until a constant weight was reached. Six replicate root samples were collected from each treatment after 30 days and washed with clean water. The main root length was measured using a steel tape measure, the root volume was measured using the drainage method, and the root activity was measured using the triphenyltetrazolium chloride method (Li, 2000).

### Determination of Leaf Gas Exchange, Photosynthetic Pigment Contents, and Chlorophyll Fluorescence Parameters

Photosynthetic indices, including net photosynthesis (Pn), stomatal conductance (Gs), intercellular CO_2_ concentration (Ci), and transpiration rate (Tr), were measured using a portable photosynthesis system machine (CIRAS-2, PP System Inc., Amesbury, MA, United States) from 9:00 am to 11 am. The chlorophyll fluorescence parameters of cucumber leaves were measured using an FMS-2 pulse-modulated fluorometer (Hansatech Instruments Ltd., Norfolk, England). FS, Fm′ under light adaptation, Fo, Fm, and Fv/Fm under dark adaptation were calculated using the formulae ΦPS II, NPQ, qP, and Fv′/Fm′ (Tang et al., 2021). The contents of photosynthetic pigments (chlorophyll a, chlorophyll b, chlorophyll a + b, and carotenoids) in fresh cucumber leaves were determined (Arnon, 1949), and each sample (0.1 g) was transferred to a 20-ml tube with 10 ml of 80% acetone and then placed in the dark for 48 h (shaken every 8 h) until the leaves turned white. Finally, the absorbance rates of the extract solution at 663 nm, 645 nm, and 440 nm were measured using a UV-1780 spectrophotometer (Shimadzu, Japan).

### Determination of Calvin Cycle Key Enzyme, Sugar Metabolism-Related Enzymes, and Photosynthate

Key enzymes of the Calvin cycle in cucumber leaves are ribulose diphosphate carboxylase/oxygenase (Rubisco), fructose-1,6-bisphosphatase (FBPase), transketolase (TK), glyceraldehyde-3-phosphate dehydrogenase (GAPDH), and fructose-1,6-bisphosphate aldolase (FBA). Sugar metabolism-related enzymes include sucrose synthase (SS), sucrose phosphate synthase (SPS), acid invertase (AI), and neutral invertase (NI). The activity of these enzymes and the contents of photosynthetic products (sucrose, fructose, glucose, and starch) were measured using an ELISA kit (Yaji Biotech, Shanghai, China).

### RNA Extraction and Quantitative Reverse Transcription-Polymerase Chain Reaction Analysis

After LED light treatment for 30 days, 10 cucumbers were selected from each treatment, and the top third leaf was removed, washed, and dried immediately and then placed in liquid nitrogen. Each replicate was 100 mg for total RNA extraction, and total RNA was extracted from the cucumber leaves using an RNA simple Total RNA Kit (Tiangen Biotech, Beijing, China) according to the manufacturer’s instructions. The EVO-M-MLV RT Kit with gDNA Clean (Accurate Biotech, Changsha, China) was used to remove the residual DNA and reverse-transcribe the extracted RNA, as recommended by the manufacturer. Quantitative reverse transcription-polymerase chain reaction (qRT-PCR) experiments were performed using an SYBR Green Premix Pro Tap HS qPCR kit (Accurate Biotech, China). Samples for qRT-PCR were obtained from three seedlings for each treatment (*n* = 3) in each qPCR test, and every sample on the sample plate contained three wells of target gene and three wells of negative control (by adding components of the reaction system except for template cDNA, and the template was replaced by RNase-free dH_2_O). The qRT-PCR was performed using the Light Cycle 96 Real-time PCR system (Roche, Switzerland), qRT-PCR was performed for 30 s at 95°C, followed by 40 cycles of 5 s at 95°C and 30 s at 60°C. The cucumber actin gene was used as an internal control according to the study described by Bi et al. (2015). The primer sequences are shown in Table 1. qRT-PCR analyses and consequent statistical data analyses were performed using the procedure described in the study by Zhao et al. (2013). Expression analyses were conducted independently three times.

**TABLE 1:** Primers used for quantitative real-time PCR assays.

Gene symbol	Accession number	Forward primer (5′→3′)	Reverse primer (5′→3′)
Actin	XM_011659465.2	CCACGAAACTACTTACAACTCCATC	GGGCTGTGATTTCCTTGCTC
*CsRbc L*	NC_007144.1	GGACAACTGTGTGGACCGAT	GACGTAGAGCACGTAGAGCC
*CsRbc S*	XM_004135046.3	CGTGTACCGTGAGAACCACA	AATCTTGGGGGCTTGTAGGC
*CsFBPase*	XM_004140842.3	GGTATTCTGTGGTGTTCGATCC	GACGTCTTCTAGGTTAGGTTC
*CsFBA*	XM_004137825.3	GCAGAGTGAGGAGGAAGCAAC	CCAAACGAGAAAGATAACGACC
*CsTK*	NM_001280671.1	ACGATGAGGTCATGAAG	CCAGCAAGATGAAGCAG
*CsGAPDH*	NM_001280743.1	CCTACCGTTGATGTCTCTGTTGTT	TTCCCTCGGACTCTTCCTTG
*CsSPS*	XM_031880474.1	CGTGATCGCAATAATAGACTA	ACAGCTACCAGTTCCGTC
*CsSS*	XM_031880140.1	GCGGAAGAGTATCTTGTAACA	ACAATATTACCGTCGCTGTAG
*CsAI*	XM_004147540.3	CTGGAATGTGGGAATGTC	GTTGAATACGAAGAGCCG
*CsNI*	XM_004144783.3	GGCGTTCGTTGGTCTATT	GGCTTGTTCTGGTGTTGC
*CsPHYA*	XM_031886782.1	CACCACCACCACAGCATACCTTC	GTGAGCATTTCGGGAGCATTTTGG
*CsPHYB*	XM_004134198.3	TTGATTGCCATGCCTCTCCAGTTC	AAGAGTTGAACCCACCAAGCAGAG
*CsCRY1*	XM_011654250.2	TGTCGGAGATGTGGCAACAAGAAG	GCAGGCTCAATGTCTTCCTCATCC
*CsHY5*	XM_004138683.3	TTCCGGGAGGGATACTGGTT	CTTGCTTGCTGTGCCGATAC

### Statistical Analysis

All experiments were performed in triplicate; the results are expressed as mean values ± standard error from three independent replicates. Data were analyzed by one-way ANOVA in SPSS version 13.00 (SPSS, Inc., Chicago, IL, United States), and treatment means were compared using Duncan’s honest significant difference test at a 0.05 level of probability (*P* < 0.05).

## Results

### Effect of Supplemental LED Light Duration on Morphological Indexes of Cucumber

**FIGURE 2:**
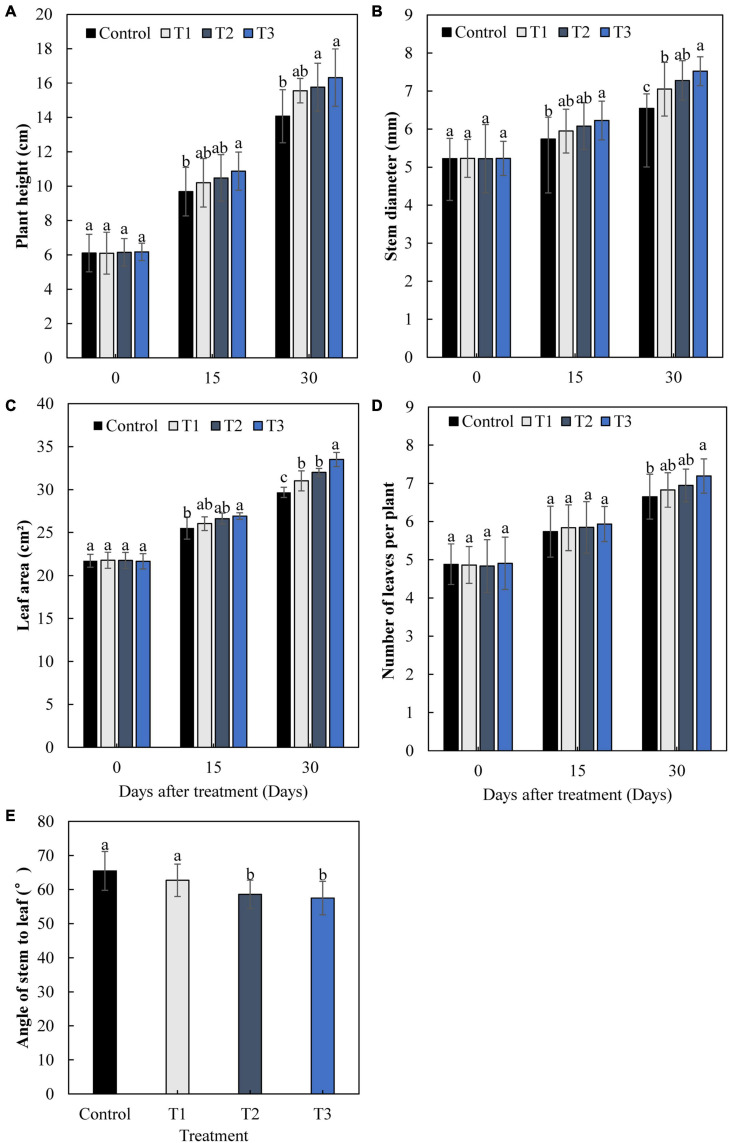
Effects of supplemental LED light duration on cucumber plant height **(A)**, stem diameter **(B)**, leaf area **(C)**, number of leaves **(D)**, and angle between stem and leaf **(E)**. Cucumber plant height, stem diameter, leaf area, and number of leaves were measured at 0, 15, and 30 days after supplemental LED light treatments. Values are expressed as the mean ± SE (*n* = 3). Bars that are significantly different based on the Duncan’s multiple range test (*p* < 0.05) are indicated with different lowercase letters.

The growth of cucumbers differed greatly with different times of LED supplementation (Figure 2). After 15 and 30 days, the plant height, stem diameter, leaf area, and leaf number of cucumbers increased gradually with an increase in light supplementation duration (Figures 2A–D). The plant height, stem diameter, leaf area, and leaf number in the T3 treatment were significantly increased by 16.0, 14.9, 12.9, and 8.2%, respectively, after 30 days, compared with those of the control. The angles between the stems and leaves of the T2 and T3 treatments were significantly lower than those of the control, with a decrease of 10.5 and 12.2%, respectively (Figure 2E). This indicates that T2 and T3 treatments had greater effects on cucumber leaves under supplemental light.

### Effect of Supplemental LED Light Duration on Root Parameters and Dry Matter Accumulation of Cucumber

The morphology and vitality of roots determine the plant growth. The main root length, root volume, and root activity of cucumbers in T2 and T3 treatments, especially T3 treatment, were significantly higher than those in the control group (Table 2). T3 treatment increased the main root length, root volume, and root activity by 22.1, 31.2, and 56.0% compared with the control group, respectively.

**TABLE 2:** Effect of supplemental LED light duration on root parameters of cucumber.

Treatment	Main-root length (cm)	Root volume (cm^3^)	Root activity (μg⋅g^–1^⋅h^–1^)
Control	29.50 ± 0.98c	3.62 ± 0.22c	97.70 ± 6.79c
T1	31.33 ± 1.53bc	4.02 ± 0.48bc	132.52 ± 7.32b
T2	33.70 ± 0.97ab	4.31 ± 0.36b	141.07 ± 4.40ab
T3	36.03 ± 2.13a	4.75 ± 0.26a	152.37 ± 8.75a

*Values are expressed as mean ± SE (*n* = 3). Bars that are significantly different based on the Duncan’s multiple range test (*p* < 0.05) are indicated with different lowercase letters.*

**FIGURE 3:**
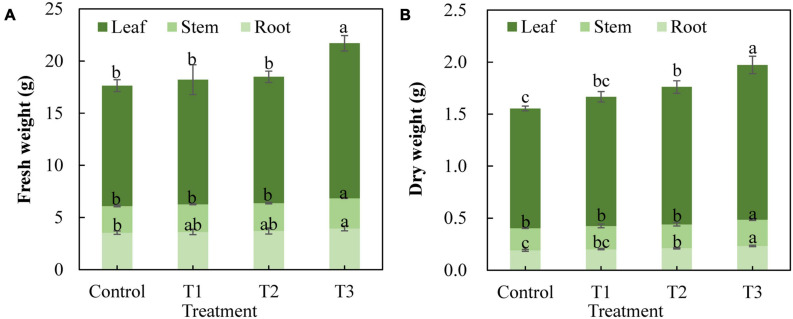
Effect of supplemental LED light duration on fresh weight **(A)** and dry weight **(B)** of cucumbers. The dry matter accumulation of cucumbers was measured on 30 days after supplemental LED light treatment. Nine cucumber plants with the same height were selected from each treatment. Values are expressed as the mean ± SE (*n* = 3). Bars that are significantly different based on the Duncan’s multiple range test (*p* < 0.05) are indicated with different lowercase letters.

Different supplemental LED light duration treatments also had an impact on the fresh weight and dry weight of cucumber plants (Figure 3). The results showed that the fresh weight of cucumber leaves, stems, and roots in the T3 treatment was significantly higher than that in the control treatment, whereas there was no significant difference between T1 and T2 treatments and the control treatment (Figure 3A). The dry weight of cucumber leaves and roots in the T2 treatment increased significantly, and the dry weight of cucumber leaves, stems, and roots in the T3 treatment increased significantly compared with that of the control treatment (Figure 3B). This indicates that T3 treatment significantly promoted the accumulation of dry matter in cucumbers.

### Effects of Supplemental LED Light Duration on Gas Exchange, Photosynthetic Pigment Content, and Chlorophyll Fluorescence Parameters in Cucumber Leaves

**FIGURE 4:**
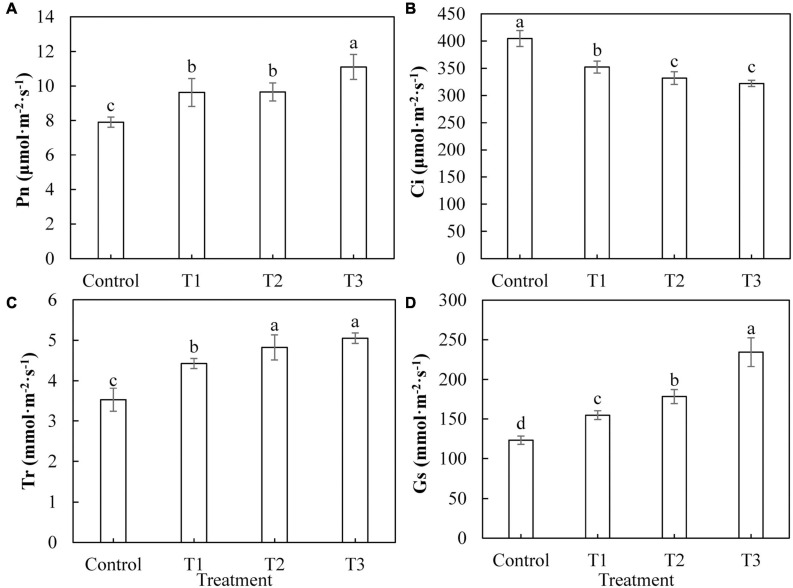
Effects of supplemental LED light duration on net photosynthetic rate **(A)**, intercellular CO_2_ concentration **(B)**, transpiration rate **(C)**, and stomatal conductance **(D)** in cucumber leaves. After supplemental LED light treatment for 30 days, the third leaf downward from the top of the cucumber was selected from 9:00 am to 11:00 am on sunny days to measure the gas exchange parameters. Values are expressed as mean ± SE (*n* = 3). Bars that are significantly different based on the Duncan’s multiple range test (*p* < 0.05) are indicated with different lowercase letters.

Figure 4 shows that photosynthesis in cucumber leaves was differently affected by supplemental LED light treatments. The net photosynthetic rate (Figure 4A), transpiration rate (Figure 4C), and stomatal conductance (Figure 4D) of cucumber leaves under T1, T2, and T3 treatments were significantly increased, compared with those of the control treatment. The increases in the T3 treatment were the most significant. The intercellular CO_2_ concentrations (Figure 4B) of cucumber leaves under T1, T2, and T3 treatments were significantly reduced compared with those of the control treatment, indicating that the photosynthesis in cucumber leaves was improved by LED supplement treatment.

As shown in Table 3, compared with the control group, the T1 treatment significantly increased the content of carotenoids in cucumber leaves. The T2 treatment significantly increased the contents of chlorophyll a, carotenoids, and total chlorophyll in cucumber leaves. The T3 treatment significantly increased the chlorophyll and carotenoid contents in cucumber leaves compared with those of the control group. These results (Table 3) indicate that LED light supplementation could increase the contents of photosynthetic pigments in cucumber leaves.

**TABLE 3:** Effects of supplemental LED light duration on photosynthetic pigment.

Treatment	Chlorophyll a (mg⋅g^–1^)	Chlorophyll b (mg⋅g^–1^)	Carotenoid (mg⋅g^–1^)	Chl (a + b) (mg⋅g^–1^)
Control	4.568 ± 0.033c	1.465 ± 0.008b	0.722 ± 0.054c	6.033 ± 0.041c
T1	4.622 ± 0.031c	1.481 ± 0.013b	0.910 ± 0.035b	6.104 ± 0.042c
T2	4.725 ± 0.062b	1.510 ± 0.064b	1.046 ± 0.032a	6.236 ± 0.064b
T3	5.125 ± 0.057a	1.635 ± 0.051a	1.052 ± 0.061a	6.760 ± 0.040a

*Values are expressed as mean ± SE (*n* = 3). Bars that are significantly different based on the Duncan’s multiple range test (*p* < 0.05) are indicated with different lowercase letters.*

**FIGURE 5:**
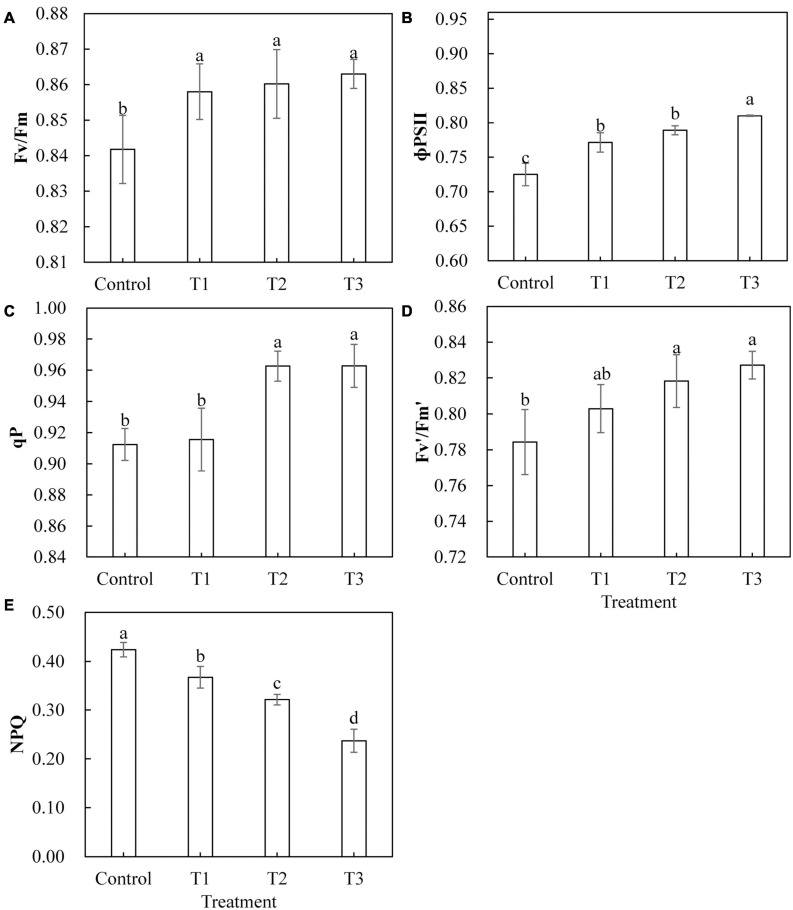
Effects of supplemental LED light duration on the maximum photochemical efficiency of PS Π **(A)**, the quantum efficiency of PS Π photochemical center **(B)**, the photochemical quenching coefficient **(C)**, the efficiency of excitation energy captured by open PSΠ reaction centers **(D)**, and the non-photochemical quenching coefficient **(E)** in cucumber leaves. After LED treatment for 30 days, chlorophyll fluorescence parameters of cucumber leaves were measured using chlorophyll fluorescence analyzer after 30 min of dark adaptation. Values are expressed as mean ± SE (*n* = 3). Bars that are significantly different based on the Duncan’s multiple range test (*p* < 0.05) are indicated with different lowercase letters.

Light-emitting diode light treatments had a significant effect on the chlorophyll fluorescence parameters of cucumber leaves (Figure 5). With the LED light treatments T1, T2, and T3, the maximum photochemical efficiency (Fv/Fm) and actual photochemical efficiency (Φ PS II) were significantly higher than those of the control (Figures 5A,B). The qP and the Fv′/Fm′ of the open PS II reaction center under T2 and T3 treatments were significantly higher than those of the control, and there was no significant difference between those of T2 and T3 (Figures 5C,D). The qP of T1 treatment and the capture efficiency of the Fv′/Fm′ of the open PS II reaction center showed no significant difference as compared to those of the control (Figures 5C,D). The non-photochemical quenching (NPQ) of T1, T2, and T3 treatments was significantly lower than that of the control treatment, and NPQ was smaller with longer LED supplementation duration (Figure 5E).

### Effects of Supplemental LED Light Duration on Calvin Cycle Key Enzyme Activities, Sugar Metabolism-Related Enzyme Activities, and Photosynthate Content in Cucumber Leaves

**FIGURE 6:**
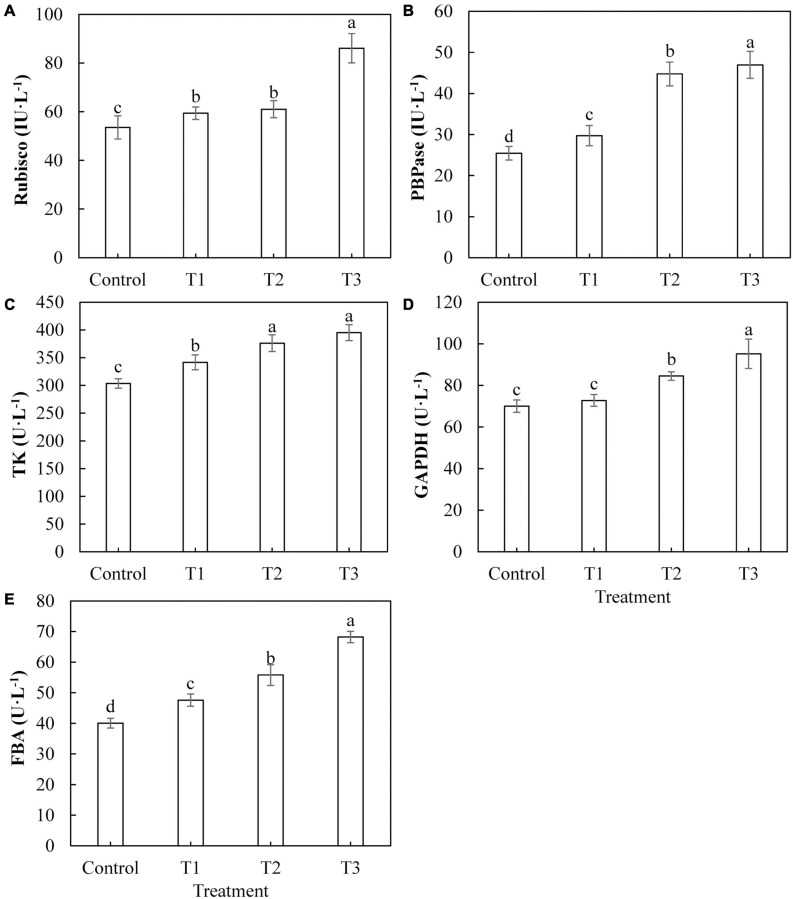
Effects of supplemental LED light duration on the activities of key enzymes of Calvin cycle: ribulose diphosphate carboxylase/oxygenase **(A)**, fructose-1,6-bisphosphonate **(B)**, transketolase **(C)**, glyceraldehyde-3-phosphate dehydrogenase **(D)**, and fructose-1, 6-bisphosphate aldolase **(E)** in cucumber leaves. After LED light supplement treatment for 30 days, 10 pieces of the third leaf from the top of cucumber were selected from each treatment, washed and dried immediately, and then put into liquid nitrogen to prepare for the determination of activities of the key enzymes in the Calvin cycle. Values are expressed as mean ± SE (*n* = 3). Bars that are significantly different based on the Duncan’s multiple range test (*p* < 0.05) are indicated with different lowercase letters.

The activities of key enzymes in the Calvin cycle directly affect the photosynthetic capacity of plants. The activities of Rubisco (Figure 6A), FBPase (Figure 6B), TK (Figure 6C), and FBA (Figure 6E) in cucumber leaves treated with LED light supplementation (T1, T2, and T3) were significantly higher than those of the control, and the activities of these enzymes showed an upward trend with an increase in LED supplementation duration. The activity of GAPDH (Figure 6D) in cucumber leaves treated with T2 and T3 was significantly higher than that of the control, whereas the activity of GAPDH in cucumber leaves treated with T1 was not significantly different from that of the control.

**FIGURE 7:**
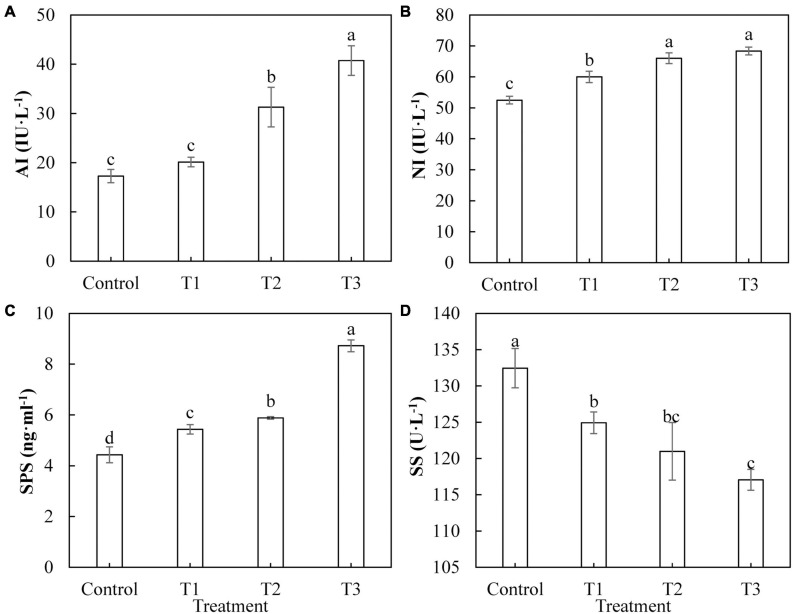
Effects of supplemental LED light duration on the activities of sugar metabolism-related enzymes: acid conversion enzyme **(A)**, neutral invertase **(B)**, sucrose phosphate synthase **(C)**, and sucrose synthase **(D)** in cucumber leaves. After LED light supplement treatment for 30 days, 10 pieces of the third leaf from the top of cucumber were selected from each treatment, washed and dried immediately, and then put into liquid nitrogen to prepare for the determination of activities of the enzymes related to sugar metabolism. Values are expressed as mean ± SE (*n* = 3). Bars that are significantly different based on the Duncan’s multiple range test (*p* < 0.05) are indicated with different lowercase letters.

Sugar is the product of photosynthesis, and sugar metabolism plays an important role in biological metabolism. As shown in Figure 7, the activities of AI (Figure 7A), Ni (Figure 7B), and SPS (Figure 7C) in cucumber leaves treated with supplemental LED, T1, T2, and T3 were significantly higher than those of the control, and the activities of these enzymes showed an upward trend with an increase in LED supplementation duration. The activity of SS (Figure 7D) in cucumber leaves under T1, T2, and T3 treatments was significantly lower than that of the control. The activity of SS under T1 treatment was the highest, whereas that under T3 treatment was the lowest.

**FIGURE 8:**
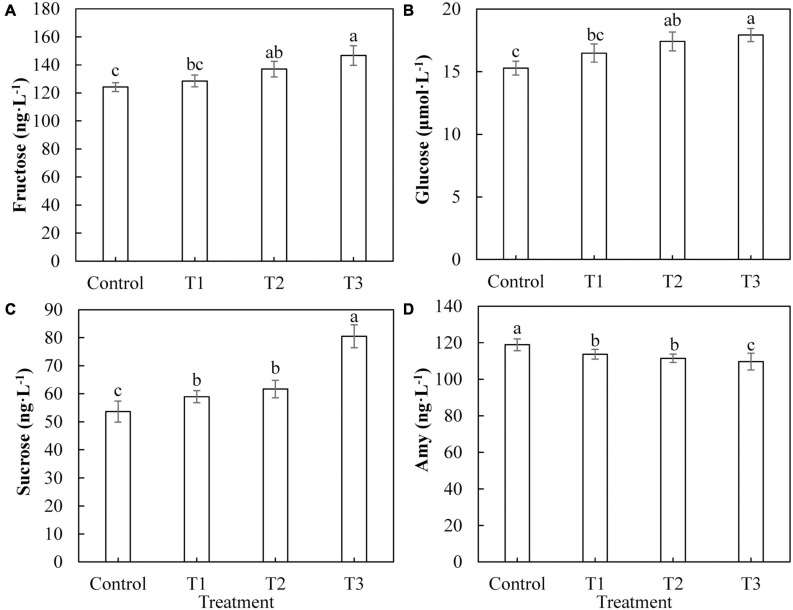
Effects of supplemental LED light duration on the contents of photosynthetic products: fructose **(A)**, glucose **(B)**, sucrose **(C)**, and starch **(D)** in cucumber leaves. After LED light supplement treatment for 30 days, 10 pieces of the third leaf from the top of cucumber were selected from each treatment, washed and dried immediately, and then put into liquid nitrogen for the determination of photosynthate contents. Values are expressed as mean ± SE (*n* = 3). Bars that are significantly different based on the Duncan’s multiple range test (*p* < 0.05) are indicated with different lowercase letters.

The most important product of photosynthesis is sugar, which provides a carbon skeleton and energy for plant growth and development. Figure 8 shows the difference in the photosynthetic product content of cucumber leaves under different supplemental LED light treatments. The contents of fructose (Figure 8A) and glucose (Figure 8B) in cucumber leaves in T2 and T3 treatments were significantly higher than those in the control group, whereas there was no significant difference between those of the T1 treatment and the control group. The sucrose content in cucumber leaves of T1, T2, and T3 treatments (Figure 8C) was significantly higher than that of the control, and the T3 treatment had the highest fructose content. The content of starch in cucumber leaves treated with T1, T2, and T3 (Figure 8D) was significantly lower than that of the control, and the content of starch in the T3 treatment was the lowest.

### Effects of Supplemental LED Light Duration on the Expression Levels of Genes Encoding key Enzymes of Calvin Cycle and Sugar Metabolism in Cucumber Leaves

**FIGURE 9:**
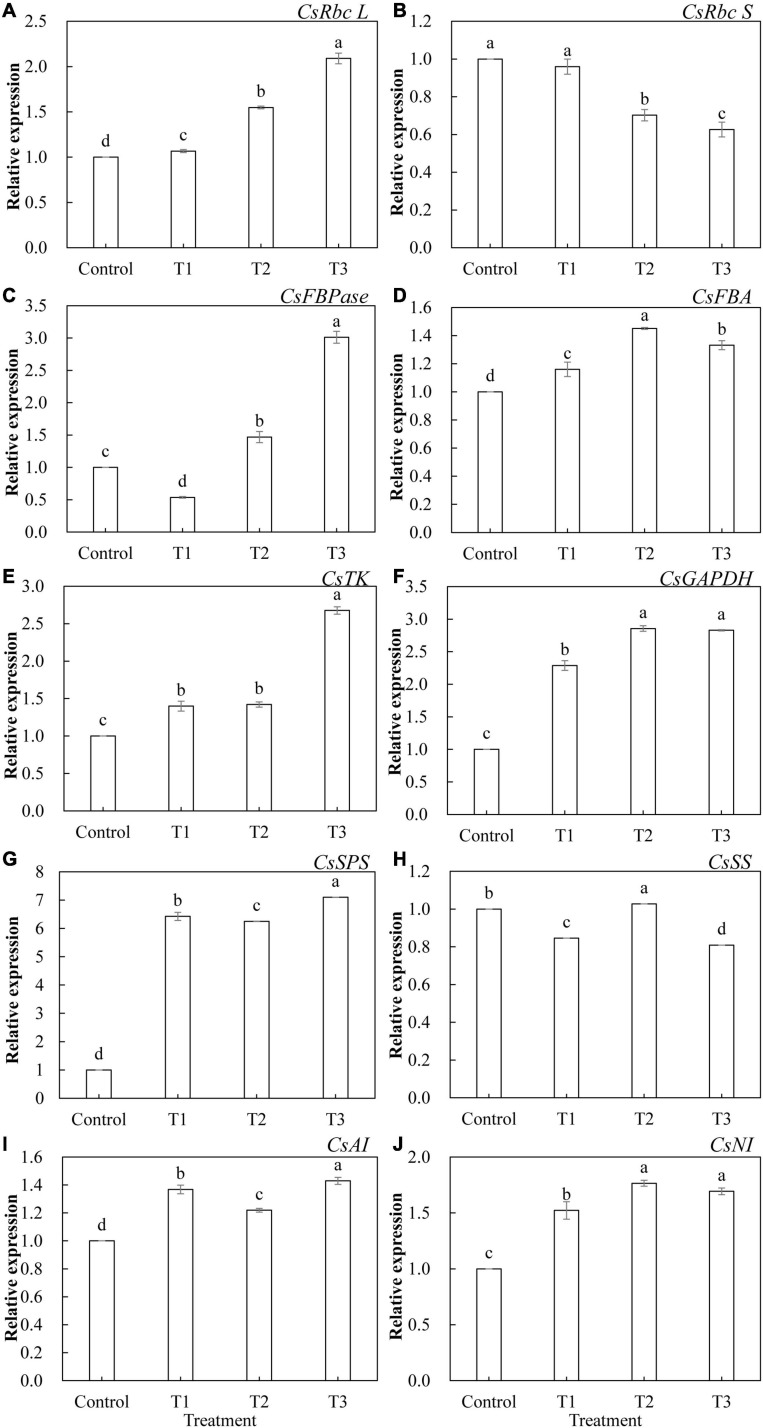
Effects of supplemental LED light duration on the expression levels of *CsRbc L* **(A)**, *CsRbc S* **(B)**, *CsFBPase* **(C)**, *CsFBA* **(D)**, *CsTK* **(E)**, *CsGAPDH* **(F)**, *CsSPS* **(G)**, *CsSS* **(H)**, *CsAI* **(I)**, and *CsNI* **(J)** in cucumber leaves. After LED light treatment for 30 days, 10 cucumber leaves were selected from the third leaf from the top in each treatment, washed and dried immediately, and then placed in liquid nitrogen. They were stored in a refrigerator at –80°C for total RNA extraction and subsequent qRT-PCR experiments. Values are expressed as mean ± SE (*n* = 3). Bars that are significantly different based on the Duncan’s multiple range test (*p* < 0.05) are indicated with different lowercase letters.

The relative expression levels of 10 genes that encode key enzymes of the Calvin cycle and related enzymes of sugar metabolism in cucumber leaves treated with different supplemental LED light durations were analyzed using qRT-PCR. As shown in Figure 9, the expression of these genes was significantly different under different supplemental LED light durations. Among the key enzyme genes of the Calvin cycle, the expression of *CsRbc L* (Figure 9A), *CsFBA* (Figure 9D), *CsTK* (Figure 9E), and *CsGAPDH* (Figure 9F) was significantly upregulated in T1, T2, and T3 treatments, while the expression of *CsRbc S* (Figure 9B) was significantly lower in the T2 and T3 treatments than in the control. The expression of *CsFBPase* (Figure 9C) gene was significantly upregulated in T2 and T3 treatments, while the expression of *CsFBPase* was significantly suppressed in the T1 treatment. Among the genes related to sugar metabolism, T1, T2, and T3 treatments significantly upregulated the expression of *CsSPS* (Figure 9G), *CsAI* (Figure 9I), and *CsNI* (Figure 9J). T2 treatment significantly upregulated the expression of *CsSS* (Figure 9H), while T1 and T3 treatments significantly inhibited the expression of *CsSS*.

### Effects of Supplemental LED Light Duration on the Expression Levels of *CsPHYA*, *CsPHYB*, *CsCRY1*, and *CsHY5* in Cucumber Leaves

**FIGURE 10:**
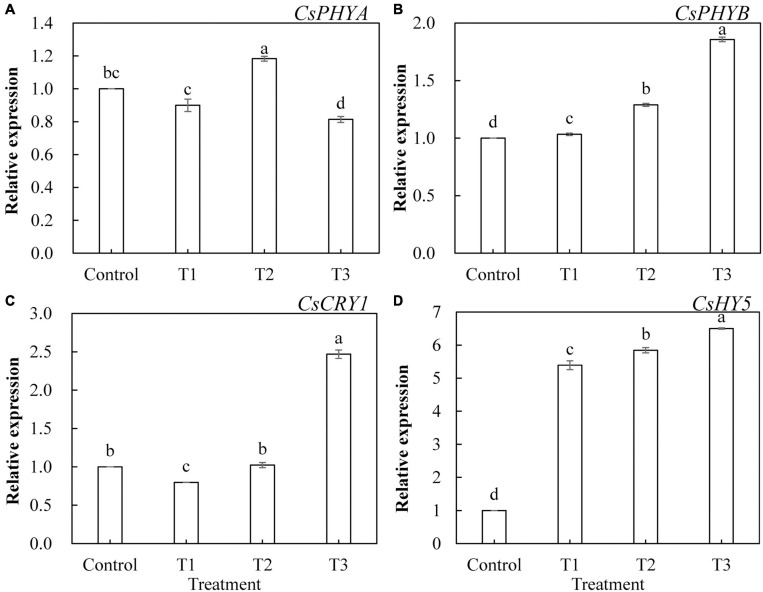
Effects of supplemental LED light duration on the expression levels of *CsPHYA* **(A)**, *CsPHYB* **(B)**, *CsCRY1* **(C)**, and *CsHY5* **(D)** in cucumber leaves. After LED light treatment for 30 days, 10 cucumber leaves were selected from the third leaf from the top in each treatment, washed and dried immediately, and then placed in liquid nitrogen. They were stored in a refrigerator at –80°C for total RNA extraction and subsequent qRT-PCR experiments. Values are expressed as mean ± SE (*n* = 3). Bars that are significantly different in terms of the Duncan’s multiple range test (*p* < 0.05) are indicated with different lowercase letters.

To further investigate the effects of LED light supplementation on phytochrome, cryptochrome, and downstream core light signal regulatory factors of phytochrome in cucumber leaves, we determined the expression of the genes encoding *CsPHYA*, *CsPHYB*, *CsCRY1*, and *CsHY5*. As shown in Figure 10, *CsPHYA* expression was significantly upregulated in T2 treatment (Figure 10A) and significantly downregulated in T3 treatment, whereas *CsPHYA* expression in T1 treatment was not significantly different from that in the control group. T1, T2, and T3 treatments significantly upregulated the expression of *CsPHYB* (Figure 10B) and *CsHY5* (Figure 10D), and the upregulated trend became more obvious with the increase in supplemental light duration. The expression of *CsCRY1* in T3 treatment was significantly upregulated (Figure 10C) and that in T1 treatment was significantly downregulated, whereas there was no significant difference between that of the T2 treatment and the control.

## Discussion

Light is one of the important factors affecting plant growth. Differences in the influence of light quality, light intensity, and photoperiod on the plant growth and development are most frequently studied. Studies have used different ratios of red and blue light (1R1B, 2R1B, and 1R2B) LEDs to treat cucumber seedlings. It was found that supplementary light significantly promoted the growth of cucumber seedlings. Among them, 2R1B seedlings showed the strongest effects, in terms of dry weight, and seedling index was high (Song et al., 2019), and supplementing with LED red and blue light (9R1B) can increase the cucumber leaf area and dry weight (Zou et al., 2020). We used LEDs that combined red and blue light (7R2B) to supplement light at the end of the day for cucumber seedlings and studied the effects of different light supplementation durations on the cucumber growth and physiology. Each supplemental light treatment showed a different effect in promoting plant height, stem thickness, leaf area, and number of leaves of cucumber seedlings after 30 days. Promotion of growth was more obvious with the increase in supplemental light duration (Figure 2). The angles between the stems and leaves of cucumber decreased as supplemental light duration increased (Figure 2E). The LED infill light in our study was placed directly above the cucumber, and the growth of plant leaves was phototropic. This indicates that as the infill light duration increased, the effect of light on cucumber leaves was increased, the phototropism of the leaves was increased, and the angle between the stems and leaves of cucumbers was decreased. Plants mainly absorb water and inorganic salts through their root systems. The stronger the plant, the more developed the root system. The dry weight of the plant can reflect the photosynthetic capacity and growth and development of the plant directly. Supplementing a certain proportion of red and blue light with LEDs significantly improved root vigor and root/shoot ratio in tomatoes and cucumbers (Paponov et al., 2020; Zhang Y. et al., 2020). Single light red, green, and blue LEDs increased the aboveground dry matter accumulation of peppers (Claypool and Lieth, 2020). In this study, the root system of cucumbers was more developed and vigorous (Table 2), and the dry matter accumulation in the upper and lower parts of the ground was greater (Figure 3) under red and blue supplemental LED light treatments. This was especially evident after 3 h of supplementary light treatment, which shows that supplemental light can promote the root growth and nutrient absorption and increase the dry matter accumulation of cucumber seedlings.

Plant photosynthesis starts with the absorption of light energy by photosynthetic pigments. The photosynthetic pigment content in plant leaves can indirectly reflect the photosynthetic capacity of plants, and gas exchange parameters can directly reflect the photosynthetic capacity of plant leaves. Red and blue light can induce the opening of plant leaf stomata and promote the absorption of CO_2_ by the leaf (Cells and Zeiger, 1983). Supplementing LED red and blue light at night in both winter and summer helps to improve the photosynthetic capacity of tomato leaves (Tewolde et al., 2016; Lanoue et al., 2019). Red and blue LEDs (1R1B) promote the growth of cherry tomato seedlings, which is related to the high content of photosynthetic pigments, the number of stomata, and the distribution of photosynthate (He et al., 2017). Our results showed that the supplemental red and blue LED light treatment significantly increased the net photosynthetic rate (Figure 4A), transpiration rate (Figure 4C), and stomatal conductance (Figure 4D) of cucumber leaves and reduced the intercellular CO_2_ concentration (Figure 4B) of cucumber leaves. This indicates that light supplementation improved the photosynthetic capacity of cucumber leaves and promoted the fixation of CO_2_ in cucumber leaves, which is consistent with the results in lettuce, tomato, and peony plants (Wang et al., 2016; Paponov et al., 2020; Wan et al., 2020). Some studies have found that red and blue (10R1B) LED treatment significantly increased the photosynthetic pigment content of tomato test-tube seedlings (Naznin et al., 2019). Our research on photosynthetic pigment content showed similar results. The contents of chlorophyll a, chlorophyll b, and carotenoids in cucumber leaves were significantly increased by light supplementation of 2 and 3 h (Table 3). This indicates that the red and blue supplemental LED light treatment may first increase the photosynthetic capacity of cucumber leaves by increasing the synthesis and accumulation of photosynthetic pigments, thereby increasing the absorption and transmission of light energy by the leaves. In chlorophyll fluorescence parameter analysis of the cucumber leaf, red and blue LED light supplementation improved the PS II maximum photochemical efficiency (Fv/Fm) (Figure 5A), the actual ΦPS II (Figure 5B), qP (Figure 5C), and open PS II reaction center excitation energy capture efficiency (Fv′/Fm′) (Figure 5D). It also reduced the coefficient of NPQ (Figure 5E). With the increase in supplemental light duration, the increasing or decreasing trend became more obvious. This is consistent with research regarding red and blue LEDs in cherry tomatoes and Chinese cabbage (Liu, 2012), which shows that supplemental light treatments can improve the degree of openness of the reaction center of the leaf photosystem II, light energy conversion efficiency, and utilization rate of plants, thereby increasing the photosynthetic capacity of the leaf.

The Calvin cycle is part of the carbon reaction in cucumber photosynthesis and is used to fix CO_2_ absorbed by the leaves. Studies have shown that different light qualities (red, blue, purple, green, and yellow) alter the plant photosynthesis by affecting the activities of Calvin cycle enzymes in plant leaves (Wang et al., 2009). We studied the activities of key Calvin cycle enzymes in cucumber leaves to explore the internal mechanisms of increased photosynthetic capacity in plants. In this study, all supplemental light treatments significantly increased the activity of Rubisco enzyme in cucumber leaves, improved the efficiency of the carboxylation stage in the Calvin cycle, and promoted the fixation of CO_2_ (Figure 6A). Supplementation with light for 2 and 3 h increased the activity of the GAPDH enzyme in cucumber leaves, increased the rate of the reduction phase, and accelerated the completion of photosynthetic energy storage (Figure 6D). All supplemental light treatments significantly increased the activities of FBPase (Figure 6B), TK (Figure 6C), and FBA (Figure 6E) in cucumber leaves, promoted the regeneration of ribulose 1, 5-diphosphate (RuBP) in the Calvin cycle, and thus accelerated the fixation of CO_2_. The results indicated that the red and blue light supplemental LED treatments improved the CO_2_ fixation efficiency by increasing the activities of Calvin cycle enzymes in cucumber leaves, thus improving the photosynthetic capacity of cucumber leaves. Sugar is the product of photosynthesis, and sugar metabolism is the center of biological metabolism. Previous studies have shown that adding blue light to red light can promote the formation of plant photosynthetic products and supplementing red and blue light (3R1B) can increase the activity of SPS and the accumulation of sucrose in tomato leaves (Li et al., 2017). Our results showed that supplemental light treatments for 2 and 3 h increased the activity of leaf acid invertase (Figure 7A), as well as the contents of fructose (Figure 8A) and glucose (Figure 8B). Supplemental light treatments for 1, 2, and, 3 h also increased the activities of leaf neutral invertase (Figure 7B) and sucrose phosphate synthase (Figure 7C) and the content of sucrose (Figure 8C). Interestingly, supplemental light treatment significantly reduced the activity of sucrose synthase (Figure 7D) and the content of starch (Figure 8D). Our results differ in this regard from the results of Li et al. (2017), which may be due to the different ratios of red and blue light of the LED used. By comparing the reactions and pathways related to sugar metabolism, we found that red and blue supplemental LED light increased the activities of SPS, AI, and NI in cucumber leaves and promoted the synthesis and accumulation of sucrose, fructose, and glucose. Fructose is also used in the reaction of SS to decompose sucrose. As the product, its large accumulation inhibits the progress of the sucrose decomposition reaction. This leads to a decrease in the SS enzyme activity, which in turn reduces the content of the direct precursor of starch, adenosine diphosphate glucose (ADPG), resulting in a significant reduction in starch content.

We further studied the expression levels of genes encoding key enzymes in the Calvin cycle and enzymes related to sugar metabolism. Previous studies have shown that the expression of the Rubisco large subunit gene in cucumbers was decreased under low light stress (Xiao et al., 2010). Overexpression of the FBA gene can promote the growth of phycocyanin bacteria under low light conditions (Liang and Lindblad, 2016). Studies on Arabidopsis have shown that overexpression of the FBPase gene can increase the photosynthetic capacity of plants and accelerate the plant growth. The combination of red and blue light (1R9B) promoted the expression of photosynthesis-related genes in *Camellia oleifera* seedlings and effectively promoted their photosynthetic capacity and growth and development (Gong, 2018). Red and blue light supplemental treatments (3R1B) promoted the expression of genes encoding enzymes related to sugar metabolism in tomato fruits (Dong et al., 2018). Our results show that the red and blue supplemental LED light treatments significantly upregulated *CsRbc L* (Figure 9A), *CsFBA* (Figure 9D), *CsTK* (Figure 9E), *CsGAPDH* (Figure 9F), *CsSPS* (Figure 9G), *CsAI* (Figure 9I), and *CsNI* (Figure 9J) expression levels. The expression level of *CsFBPase* (Figure 9C) was significantly upregulated with 2 and 3 h of light supplementation, and the gene expression level showed an upward trend with the increase in the duration of light supplementation. This was consistent with the changes in the activities of the key enzymes in the Calvin cycle. However, after 1 h of supplemental light, the expression level of *CsFBPase* downregulated compared with the control. The reason might be that at the beginning of supplementary light, a large amount of photosynthetic products have already been synthesized and accumulated in cucumber leaves during daytime; these photosynthetic products could not be exported from the plastid in time, then, indirectly inhibited the production of fructose 6-phosphate (the precursor of sucrose) through a negative feedback role. And fructose 6-phosphate is produced from fructose 1,6-diphosphate catalyzed by FBPase, so it indirectly led to the decrease in FBPase activity. Therefore, the expression level of *CsFBPase* was inhibited when the light was supplemented for 1 h (Pang et al., 1997; Cheng et al., 2004; Luo, 2004). The expression of *CSRbc S* in cucumber leaves showed a decreasing trend (Figure 9B), with an increase in the duration of light supplementation, compared with that of the control. This may be caused by the negative correlation between the expression of *CsRbc S* and the content of Rubisco (Suganami et al., 2018). The expression of *CsSS* (Figure 9H) in cucumber leaves showed a trend of first increasing and then decreasing with the increase in light supplementation duration. It may be that light supplementation in the early stages increased the photosynthetic ability of cucumber and then upregulated the expression of *CsSS* with the large accumulation of fructose in the leaves; the ability of the SS enzyme to decompose sucrose was inhibited. Plants thus reduce the decomposition reaction of the SS enzyme by downregulating the expression of *CsSS* to balance the photosynthetic products inside the plant. This is consistent with the results of the research regarding the SS enzyme activity. These results indicate that the supplementation of red and blue LED light increased the activities of most of the Calvin cycle key enzymes and the sugar metabolism-related enzymes by upregulating the expression levels of most of the genes encoding these enzymes, thus improving the photosynthetic capacity of cucumber leaves and promoting the synthesis and accumulation of photosynthates.

**FIGURE 11:**
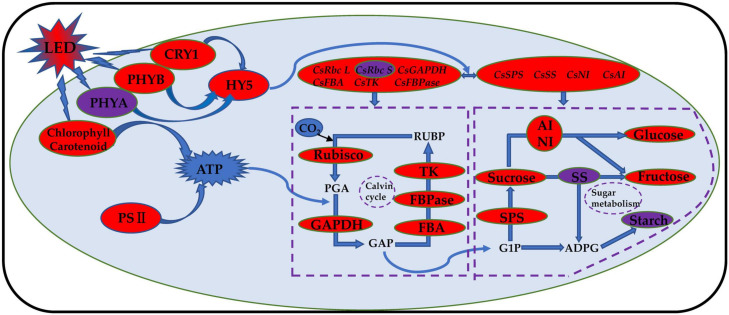
Schematic model of photosynthesis response of cucumber to red and blue LED supplemental light. The red and blue LED light supplement can improve the photosynthetic capacity of cucumber and promote the accumulation of photosynthetic products by regulating the transcriptional level and activity of photoreceptors, core light signal regulators, Calvin cycle enzyme, and glucose metabolism enzyme; improve the photosynthetic pigment content; and promote the opening of the reaction center of cucumber leaf photosystem II. The increase is indicated by the red oval. The decrease is indicated by the purple oval.

Phytochrome (PHY) is sensitive to red light, and cryptochrome (CRY) is sensitive to blue light. The phytochrome family can respond to different ratios of red and far-red light, and PHYB can react to a low ratio of red to far-red light. PHYA can only play a role when the red and far-red light ratios are less than 0.3 (Smith et al., 1997; Smith, 2000; Lau and Deng, 2012). Previous studies have shown that the mechanism of tomato stem elongation in a low ratio of red to far-red light is mediated by PHYA, PHYB, and CRY1 and that red and blue LED irradiation increases the levels of HY5, PHYA, and PHYB in tomato fruits (Xie et al., 2019). Studies on blueberries have shown that the photoreceptor genes PHYA and PHYB have the highest expression levels under red and blue (6R1B) LED treatment, while the photoreceptor gene CRY1 has the highest expression level under red and blue (3R1B) LED treatment (Wang, 2020). Compared with red light, a low ratio of red to far-red light treatment induced an increase in leaf area and stem elongation of tomatoes by inhibiting HY5 protein accumulation (Zhang, 2019). Our results showed that the expression levels of *CsPHYB* (Figure 10B) and *CsHY5* (Figure 10D) in cucumber leaves were significantly upregulated by light supplementation compared with those of the control treatment and showed an upward trend with the increase in supplemental light duration. This showed the same trend as that of the expression levels of key enzyme genes in the Calvin cycle. *CsPHYA* expression in the cucumber leaf (Figure 10A) first increased and then decreased with increasing supplemental light duration. This may be because there is a small amount of far-red lights activated the far-red receptor PHYA at first PHYA will be destabilized by E3 ubiquitin ligases, including COP1, under large amounts of red light irradiation, promoting the degradation of PHYA (Li et al., 2002; Saijo et al., 2008; Rausenberger et al., 2011). When light supplementation commenced, the expression of *CsCRY1* in cucumber leaves (Figure 10C) was significantly downregulated as compared to that of the control. However, with an increase in light supplementation duration, the expression of *CsCRY1* gradually increased, which may be due to dark conditions; the COP1/SPA complex recognizes and ubiquitinates HY5 and then degrades the HY5 protein through the 26S proteasome. The cryptochrome protein structure changes after exposure to light, and the CRY1 protein interacts with the SPA1 protein in a blue light-dependent manner through its CCT1 domain, to separate the SPA1 and COP1 protein. The expression of *CsCRY1* in cucumber leaves will thus be downregulated at the beginning of light supplementation. The tight binding between COP1 and SPA1 proteins provides a crucial condition for COP1 to exert its E3 ubiquitin ligase activity. After light supplementation commences, blue light causes the COP1/SPA complex to dissociate, and the E3 ubiquitin ligase activity of COP1 is inhibited (Wang, 2017). Eventually, transcription factor proteins such as HY5 are prevented from degradation, and the CRY gene will no longer interact with SPA1. The proteins interact; therefore, with the increase in light supplementation duration, the expression of *CsCRY1* in cucumber leaves will gradually increase (Osterlund et al., 2000; Wang et al., 2001; Lian et al., 2011; Liu et al., 2011). Based on the above results, we found that red and blue (7R2B) LED end-of-day supplemental light may activate the expression of PHYB and CRY1 genes, thereby promoting the downstream core optical signal regulating HY5 transcription and accumulation. This activates the transcription of genes related to the key enzymes of the Calvin cycle and sugar metabolism, improves the photosynthetic capacity of cucumbers, increases the accumulation of photosynthetic products, and promotes the growth of cucumber seedlings (the response path and regulation mode are shown in Figure 11).

## Conclusion

Our research showed that using LEDs to supplement red and blue light (7R2B) for 3 h significantly promoted the growth and development of the cucumber plant; enhanced the root growth and dry matter accumulation; and improved the photosynthetic rate, photosynthetic pigment content, and light energy utilization efficiency of cucumbers. Red and blue light supplementation using LEDs for 3 h increased the enzyme activity and promoted the synthesis and accumulation of photosynthetic products. This was achieved by upregulating the expression of the genes encoding the key enzymes of the Calvin cycle and sugar metabolism-related enzymes in cucumber leaves; this process may be regulated by PHYB, CRY1, and HY5. In conclusion, we showed in this experiment that using LEDs to supplement red and blue light for 3 h is appropriate for cucumber cultivation in greenhouses in winter. These results provide a theoretical reference for the design of LED light supplementation methodologies for other plants.

## Data Availability Statement

The original contributions presented in the study are included in the article/supplementary material, further inquiries can be directed to the corresponding author/s.

## Author Contributions

JY and JL conceived and designed the research. SW and HF conducted the experiments. SW, HF, JL, and YW analyzed the data and prepared the figures and illustrations. ZT and ZL contributed to the materials. SW wrote the manuscript. JL, JY, HF, and JX read the manuscript and made valuable inputs. All authors read and approved the submission of the manuscript.

## Conflict of Interest

The authors declare that the research was conducted in the absence of any commercial or financial relationships that could be construed as a potential conflict of interest.
